# Cerebral Microstructural Alterations after Radiation Therapy in High-Grade Glioma: A Diffusion Tensor Imaging-Based Study

**DOI:** 10.3389/fneur.2017.00286

**Published:** 2017-06-15

**Authors:** Rebecca Kassubek, Martin Gorges, Mike-Andrew Westhoff, Albert C. Ludolph, Jan Kassubek, Hans-Peter Müller

**Affiliations:** ^1^Department of Neurology, University of Ulm, Ulm, Germany; ^2^Department of Pediatrics and Adolescent Medicine, University Medical Center Ulm, Ulm, Germany

**Keywords:** radiation therapy, fractional anisotropy, diffusion tensor imaging, glioma, magnetic resonance imaging

## Abstract

**Objective:**

To investigate radiation therapy-induced microstructural damage of white matter in patients with high-grade glioma by diffusion tensor imaging (DTI).

**Methods:**

DTI was performed in 18 patients with high-grade glioma (WHO grades III and IV) and 13 healthy controls. DTI images were cross-sectionally aligned for the calculation of baseline fractional anisotropy (FA). Interhemispheric FA values in patients with high-grade glioma before or without brain radiation therapy were compared with the interhemispheric FA values in patients after radiation therapy and in healthy controls. In a subgroup without any clinical or diagnostic evidence of tumor progression, serial DTI data (5–11 scans) before and after radiation therapy were collected and longitudinal interhemispheric FA changes were assessed and compared to longitudinal data from the control group.In addition, interhemispheric axial, mean, and radial diffusivity was assessed.

**Results:**

Global interhemispheric FA reductions could be detected cross-sectionally in patients after radiation therapy; these were significantly different from global interhemispheric FA differences both in patients without radiation and in healthy controls. Longitudinal scans in patients with radiation therapy confirmed these findings and revealed progressive microstructural white matter damage after partial brain radiotherapy. The additional DTI metrics axial diffusion, mean diffusivity, and radial diffusion confirmed interhemispheric differences in patients without or before radiation therapy, which were lower than the differences in patients after radiation therapy, although not reaching significance.

**Conclusion:**

Interhemispheric global FA differences could potentially serve as a biological marker for irradiation-induced microstructural white matter damage.

## Introduction

High-grade glioma, including glioblastoma as the most common primary brain tumor, account for approximately 25% of all central nervous system tumors. Despite multidisciplinary treatment approaches, the prognosis of high-grade glioma is still poor, and median survival of patients with glioblastoma is only 15 months, although patients with primary brain tumors of WHO grade III exhibit longer survival rates of up to several years (1). At present, the standard of care for the treatment of newly diagnosed high-grade glioma after maximal safe surgical resection is based on radiation therapy or chemotherapy, either alone or as combined radio-/chemotherapy (2). Although cranial radiation therapy leads to a significant benefit with respect to overall survival in patients with primary brain tumors (3), it is also associated with some substantial disadvantages with respect to quality of life. Irradiation-induced disabling cognitive impairments occur in 50–90% of patients who survived for more than 6 months after radiation therapy, mainly in those who received whole brain radiation therapy and are also found after partial brain radiation therapy (4–6). Central nervous system dysfunction is assumed to be due to early irradiation-induced inflammation as well as loss of brain structural integrity. The latter might be associated with chronic inflammation and degradation of progenitor cell niche, which potentially causes progressive neuronal loss and, therefore, could explain long-term cerebral dysfunction after radiation therapy (7, 8). The assessment of this damage with routine neuroimaging tools is limited, whereas diffusion tensor imaging (DTI) allows for the analysis of the structural connectivity and has already shown to be a non-invasive tool to probe white matter integrity, identifying white matter microstructural changes at a higher sensitivity than by T1- and T2-weighted magnetic resonance imaging (MRI) (9). DTI as an application of diffusion weighted imaging, which is sensitive to the random Brownian motion of water molecules, uses parameters such as fractional anisotropy (FA), representing the directionality of water diffusion, the mean diffusivity (MD), the radial diffusion (RD), and the axial diffusion (AD) (10). To date, only a handful of studies have addressed the non-invasive identification of irradiation-induced microstructural CNS damage in which white matter changes have been reported either very early after radiation therapy (11) or up to 11 months afterward (12). In another study, radiation-induced damage of elongated white matter bundles was assessed by the additional use of automated WM tractography analysis (13), while Chapman and co-workers investigated changes in DTI at the end of radiation therapy of low-grade tumors as a predictor for cognitive decline (14). With respect to the long-term impairment after radiation therapy, longitudinal studies are necessary to evaluate the progressive effects on white matter after radiation therapy. However, with regard to the aggressive course of WHO grade IV glioma in particular, it might be difficult to distinguish the effects on white matter and cognitive changes mediated by radiation therapy from those due to tumor progression. Therefore, to avoid bias due to tumor recurrence, only patients without any evidence for tumor progression were included in our long-term study. In detail, we aim to identify microstructural changes only due to radiation therapy (and not due to the tumor) by the analysis of peritumoral FA values (15, 16) and how they proceed over time, i.e., patients with any signs of tumor progression were thus not included in the study. We used cross-sectional and longitudinal DTI measurements to calculate FA, AD, MD, and RD differences, thereby enabling us to individually screen the possible damage to the structural integrity of the brain over time.

## Materials and Methods

### Subjects and Inclusion Criteria

The subjects’ characterization and the scan statistics are summarized in Table 1. Eighteen patients with high-grade glioma (WHO grades III and IV) and 13 healthy matched controls were included in this retrospective study. Patients were 9 males and 9 females (mean age 56 years, range 34–75), while controls were 7 males and 6 females (mean age 54 years, range 19–78; *p* = 0.77 for age differences and *p* = 0.84 for gender differences). All patients had the histologically confirmed diagnosis of high-grade glioma (anaplastic oligodendroglioma, anaplastic astrocytoma, anaplastic oligoastrocytoma, glioblastoma). Nine patients had undergone complete tumor resection, one had partial resection, and eight had stereotactic biopsy. With respect to the longitudinally assessed patients, there was no clinical or diagnostic evidence of tumor progression within the time period of the study. None of the healthy controls had a history of neurological or psychiatric disease or other medical condition, none had been diagnosed with cancer of any entity, and none of them had undergone cranial radiation therapy. All subjects gave written informed consent for MRI acquisition in accordance with the Declaration of Helsinki. The local ethics committee of the University of Ulm approved the study (project #41/17).

**Table 1:** Clinical data of the patients including scan statistics.

Patient	Scans	Scans without radiation therapy	Site of tumor	Histological diagnosis
1	1	0	Left temporal lobe	Glioblastoma
2	1	1	Right temporal lobe	Glioblastoma
3	1	0	Left frontal, temporal lobe	Anaplastic astrocytoma
4	1	1	Left frontal, parietal, temporal lobe	Glioblastoma
5	1	0	Left occipital lobe	Anaplastic astrocytoma
6	1	0	Right parietal lobe	Glioblastoma
7	1	1	Left parietal lobe	Anaplastic oligodendroglioma
8	1	0	Left frontal lobe	Anaplastic oligoastrocytoma
9	2	2	Left parietal lobe	Glioblastoma
10	2	1	Left frontal, parietal lobe	Anaplastic oligodendroglioma
11	2	0	Left temporal lobe	Anaplastic astrocytoma
12	2	2	Left frontal, temporal lobe	Anaplastic astrocytoma
13	2	0	Left frontal lobe	Anaplastic astrocytoma
14	2	2	Right temporal lobe	Glioblastoma
15	4	0	Left frontal lobe	Anaplastic astrocytoma
16	11	1	Right temporoparietal lobe	Anaplastic astrocytoma
17	10	2	Left postcentral reg.	Anaplastic oligoastrocytoma
18	5	1	Right temporal lobe	Glioblastoma
	50	12		

### DTI Scanning Protocol

Diffusion tensor imaging data sets from 18 patients and 13 healthy matched controls were recorded with the identical scanning protocol. DTI scanning was performed on a 1.5 T Magnetom Symphony (Siemens Medical, Erlangen, Germany) consisting of 52 volumes (64 slices, 128 × 128 pixels, slice thickness 2.8 mm, pixel size 2.0 mm × 2.0 mm) representing 48 gradient directions (*b* = 800 s/mm^2^) and four scans with gradient 0 (*b* = 0). The echo time and repetition time were 95 and 8,000 ms, respectively.

Longitudinal scans were available for 9 patients and for all 13 controls; the average time interval between the scans of the studied population was 24 months (±11, range 2–111 months). In summary, 50 scans from patients were recorded, of which 12 were recorded either before or without radiation therapy, while 38 scans were recorded after radiation therapy (Table 1).

### Data Analysis

The DTI analysis software Tensor Imaging and Fiber Tracking (17) was used for data processing.

#### Preprocessing: Alignment of Individual Longitudinal Scans and FA Calculation

In a first step, b0 maps (recordings with no diffusion encoding gradients) of longitudinal scans were half-way aligned (18) to the b0 map of the baseline scan for all individuals separately by a conjugate simplex fitting procedure, thus minimizing the intensity differences. In a second step, an average b0 map was calculated for each individual from all aligned b0 maps, and then all b0 maps (including the baseline scan) were aligned to that b0 template. Thus, the bias of the baseline scan was minimized. Spatial alignment of all scans was performed by rigid brain transformation to the AC-PC line. For these spatially aligned scans, FA maps were calculated by

(1) $$\text{FA}=\frac{\sqrt{{\left(\lambda_{1}-\lambda_{2}\right)}^{2}+{\left(\lambda_{1}-\lambda_{3}\right)}^{2}+{\left(\lambda_{3}-\lambda_{2}\right)}^{2}}}{\sqrt{2(\lambda_{1}^{2}+\lambda_{2}^{2}+\lambda_{3}^{2})}}$$

For more details of DTI data analysis, refer to Ref. (19, 20).

#### Post-Processing: Region of Interest (ROI) Analyses

**Figure 1:**
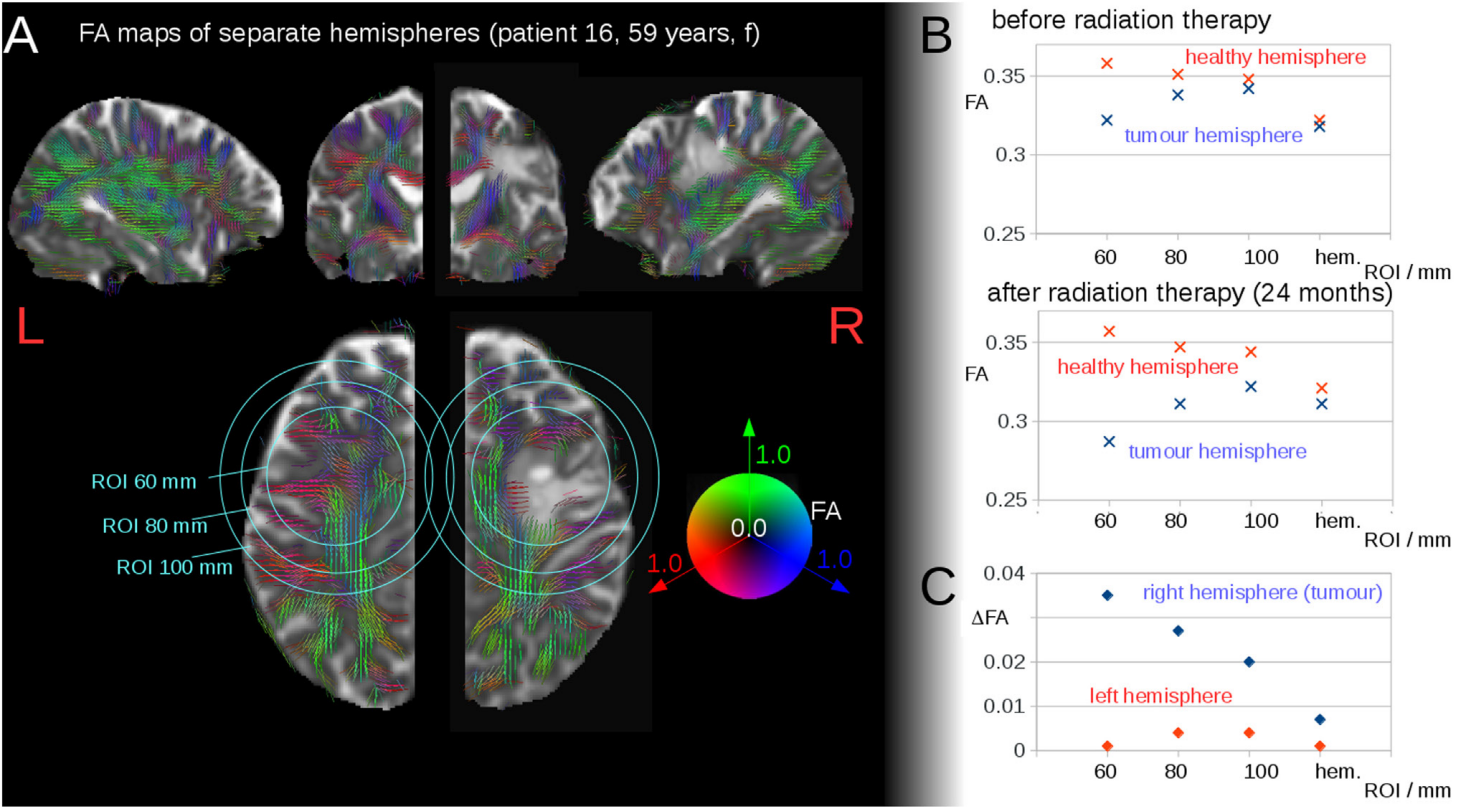
**(A)** Region of interest (ROI) analyses of fractional anisotropy (FA) around the tumor region, in one exemplary patient (patient 16). FA values are displayed as color-coded lines illustrating the main axis of the diffusion tensor. Representative slices (axial, coronal, sagittal) centered on the center-of-mass of the tumor. **(B)** Interhemispheric FA values for the ROIs and the hemisphere (hem.) before radiation therapy and after radiation therapy. **(C)** Interhemispheric FA differences (ΔFA) before radiation therapy and after radiation therapy.

Regions of interest (ROIs) with varying radius were defined around the center-of-mass of the tumor. Average FA values were calculated in these ROIs using an FA threshold of 0.2 [gray matter and tumor tissue show FA values <0.2 (21)]; the average FA values were calculated for each hemisphere separately so that FA values of the contralateral hemisphere were not incorporated in the hemisphere-wise analyses (Figure 1A). A quality check was performed on T2-weighted scans with FA maps in order to ensure that no tumor tissue contributed to the ROI-averaged FA value at an FA threshold of 0.2. These ROI-averaged FA values were referred to as “local” in the following, and the corresponding differences between the ROIs in the contralateral hemispheres were defined as local interhemispheric differences. Additionally, the average value over the whole hemisphere was calculated, leading to the definition of global interhemispheric FA differences (ΔFA) by calculating the difference of average FA values of the healthy hemisphere and average FA values of the hemisphere with the tumor.

Regions of Interest were used instead of a segmentation of the T2 lesion, as by DTI-based FA-thresholding [FA <0.2, see also Section “Post-Processing: Region of Interest (ROI) Analyses”], it was assured that no tumor tissue voxels contribute to the average FA calculation. Thus, FA values were obtained from a certain distance from the tumor center, and only white matter contributions to the averaged FA values were accounted for.

#### Post-Processing: Cross-sectional Analysis

Differences in global FA values (global interhemispheric differences—ΔFA) between both hemispheres were calculated individually for all patients (baseline and follow-up) and for controls. The statistical comparison of these interhemispheric differences (ΔFA) at the group level was performed by Wilcoxon–Mann–Whitney test.

#### Post-Processing: Longitudinal Analysis

Global interhemispheric differences (ΔFA), calculated for the entire hemisphere, were correlated with the time interval to the radiation therapy (for values before radiation therapy, the time interval was set to 0). ΔFA was also calculated for the three patients with follow-up scans and correlated with the time interval to the radiation therapy. Spearman’s rank correlation was used for calculation of the global interhemispheric FA reductions for all DTI scans of all patients, with the time interval Δ*t* to the radiation therapy.

Subgroup analyses among different tumor types and different tumor grades revealed no significant results, mostly due to the low number of members in each subgroup.

#### Post-Processing: Voxelwise Statistical Comparison for Patients before and after Radiation Therapy

The intra-subject comparison analysis of patients’ data before and after radiation therapy followed the procedures described in detail previously (19) and was adapted for intra-subject analysis. Here, after alignment of all FA maps, whole brain-based spatial statistics (WBSS) was performed by analyzing one group of data recorded before radiation therapy and the second group of data recorded after radiation therapy. Spatially aligned FA maps underwent a Gaussian smoothing filter of 8 mm FWHM since the filter size of 8 mm is about 2–3 times the recording voxelsize and provides a good balance between sensitivity and specificity (22). Then, voxelwise statistical comparisons (WBSS) were performed between the scans before and after radiation therapy by Mann–Whitney test with correction for multiple comparisons using the false-discovery-rate (FDR) algorithm (23) at *p* < 0.05 and a clustering procedure at a threshold cluster size of 512 voxels to reduce type I and type II errors (19, 24).

## Results

### Interhemispheric FA Differences—Cross-sectional Analysis

Region of Interest-based analyses of FA surrounding the tumor region showed interhemispheric FA reduction (depending on the ROI radius), caused by the tumor itself as well as by brain deformations resulting from the tumor extension (Figures 1A,B). Figure 1B illustrates the FA development for the ROI analyses of one patient as a function of the ROI radius before and after radiation therapy. The FA values in the ROIs in the tumor hemisphere were lower compared to those in the contralateral hemisphere (local interhemispheric differences). However, these differences were not present at the interhemispheric level for patients without or prior to radiation therapy (cross-sectional analysis) (single patient example is shown in Figure 1B, upper graph). For patients after radiation therapy, a longitudinal interhemispheric FA difference (ΔFA) remained (Figure 1B, lower graph). These interhemispheric (intrasubject) differences showed an FA reduction (ΔFA) for the whole tumor hemisphere (Figure 1C). These global interhemispheric differences (or FA reductions) are referred to as interhemispheric differences/reductions below.

**Figure 2:**
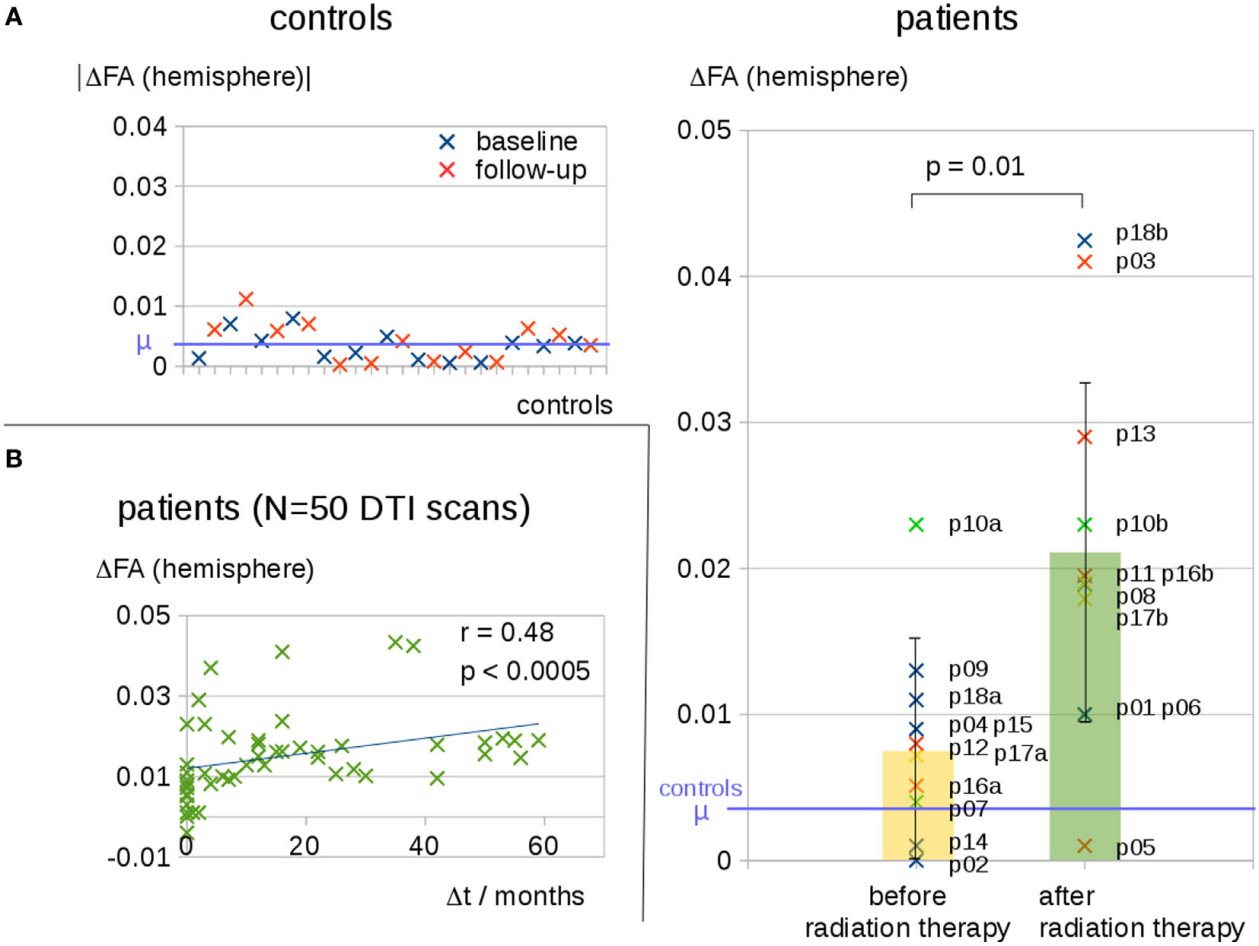
**(A)** Interhemispheric fractional anisotropy (FA) differences for controls at baseline and at follow-up and cross-sectional comparison of 10 patients before or without radiation therapy and 11 patients after radiation therapy. Distinct tumor entities were color coded: blue, glioblastoma; red, anaplastic astrocytoma; green, anaplastic oligodendroglioma; yellow, anaplastic oligoastrocytoma. **(B)** Interhemispheric FA differences ΔFA for all 50 diffusion tensor imaging (DTI) scans of all patients correlate to the time interval Δ*t* to the radiation therapy—Δ*t* of scans before radiation therapy was set to 0.

This finding encouraged us to further investigate the interhemispheric FA reduction in patients after radiation therapy compared to interhemispheric FA reduction in patients before radiation therapy and healthy controls. Control scans defined a noise level of ΔFA = 0.005 for interhemispheric FA differences; this noise level included FA reduction as well as FA increase. Patients without or before radiation therapy exhibited interhemispheric FA reductions that were significantly lower than the interhemispheric FA reductions for patients after radiation therapy. The cross-sectional comparison of the patients before or without radiation therapy (mean ΔFA ± SD, 0.007 ± 0.008) with the patients after radiation therapy (mean ΔFA, 0.021 ± 0.013) showed a statistically significant difference, *p* = 0.01 (Figure 2A). NB: three patients were included in both groups, one time before and one time after radiation therapy; only one scan before or after radiation therapy contributed to this analysis for each patient.

The additional DTI metrics, i.e., axial, mean, and radial diffusivity, revealed interhemispheric differences between patients without or before radiation therapy that were lower than the differences for patients after radiation therapy; however, these differences did not reach statistical significance (Figure S1 in Supplementary Material).

### Interhemispheric FA Differences—Longitudinal Analysis

**Figure 3:**
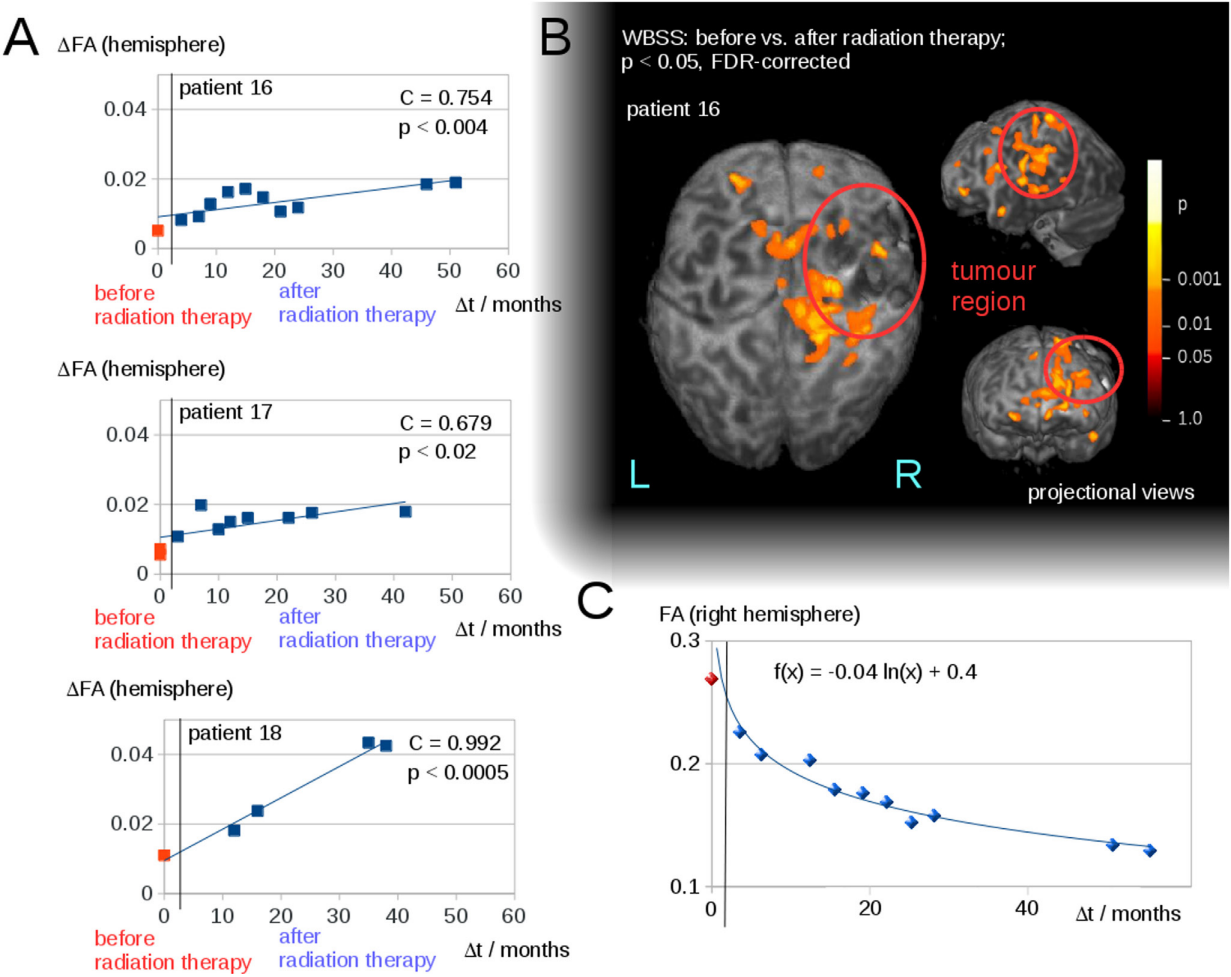
**(A)** Interhemispheric fractional anisotropy (FA) differences ΔFA for longitudinal scans of the three patients with a minimum of five scans. **(B)** Whole brain-based spatial statistics (WBSS) of patient 16, comparing the FA maps before and after radiation therapy (*p* < 0.05, FDR-corrected)—projectional views. **(C)** Longitudinal course of averaged FA values over time after radiation therapy (solid line).

Interhemispheric FA reductions (calculated for the entire hemisphere) for all 50 DTI scans of all patients correlated significantly with the time interval Δ*t* to radiation therapy (*C* = 0.48, *p* < 0.0005); Δ*t* of the scans before radiation therapy was set to 0 (Figure 2B). For the longitudinal scans of individual subjects, a significant correlation was observed between the interhemispheric FA reductions, ΔFA, and the time interval Δ*t* to the radiation therapy (Figure 3A).

### Voxelwise Statistical Comparison for Patients Prior to and after Radiation Therapy

The WBSS comparison between the scans before and after radiation therapy (Student’s *t*-test, FDR-correction at *p* < 0.05) showed significant differences, adjacent to the tumor area (which could be caused by tumor-initiated brain deformations), as well as in brain areas distinct from the tumor. These latter areas were, however, located mainly in the ipsilateral hemisphere (for an example, Figure 3B). As a complementary illustration, the changes over time in hemispherically averaged FA values (tumor hemisphere of patient 16) were visualized (Figure 3C).

## Discussion

This neuroimaging study aimed to investigate *via* DTI whether partial brain radiation therapy induces alterations of white matter integrity and how these potential alterations might progress over time. There is some evidence that DTI may help in distinguishing between high-grade glioma and brain metastases by the analysis of peritumoral FA and MD values (15) or may help in differentiating tumor progression from pseudoprogression (16). On the other hand, DTI is a possible tool to improve radiation therapy target delineation as it has been suggested that DTI detects white matter changes due to tumor tissue itself earlier than standard MRI (25). With respect to the aim of this study, it has been previously reported that DTI might serve as a tool to assess early radiation-induced injuries to normal-appearing white matter in high-grade glioma patients (11). However, so far, no long-term data or comparisons between patients with high-grade glioma with and without radiation therapy have been available. Therefore, we attempted in the current study to identify microstructural changes only due to radiation therapy (and not due to the tumor) and to assess progression of microstructural white matter changes by longitudinal measurements. Given the distinct tumor sites and the remaining tumor mass after resection or biopsy, we chose to measure interhemispheric FA differences (9).

To avoid substantial bias with respect to the differentiation of radiation-induced versus tumor-associated changes, we assessed interhemispheric FA differences in a first cross-sectional step. Here, it could be demonstrated that differences in FA reductions were not due to the tumor mass itself, since alterations of FA due to tumor itself and tumor-induced deformations of the surrounding brain structure dissolved at the interhemispheric level in patients without or before radiation therapy. In addition, patients without or before radiation therapy showed significantly lower interhemispheric FA differences than those of the patients after brain radiation therapy, thus suggesting that interhemispheric FA reductions seemed to be an appropriate parameter to assess radiation therapy-induced white matter damage and that this approach may serve as a non-invasive biological marker for irradiation-induced damage to the white matter. Furthermore, by setting the threshold FA values at 0.2—given that tumor tissue has FA values of <0.2—only white matter is taken into account for average FA calculations. Thus, ROI-based FA calculations directly reflect the integrity of white matter structures independent of the tumor size (see also visualization of thresholded FA maps in Figure 1).

From cross-sectional and longitudinal data, a significant correlation between interhemispheric FA reductions and the time interval after radiation therapy could be observed, since, even after more than 5 years after partial brain radiation therapy, there was still a progressive damage in white matter integrity. This is probably due to the degradation of the progenitor cell niche or chronic inflammation after radiation therapy that may cause progressive neuronal cell loss (8). These factors are considered to be associated with the clinical deterioration of patients with primary brain tumors without any evidence of tumor progression. Thus, DTI may allow us to distinguish between clinical deterioration due to either tumor progression or microstructural damage of white matter integrity. While FA is sensitive to microstructural changes and a summary measure of microstructural integrity, AD is sensitive to axonal injury, RD is sensitive to demyelination, and MD is a global measure of diffusivity. Thus, each of the DTI metrics could potentially serve as a marker; however, FA is the most sensitive, generalized parameter to be changed in global integrity changes of white matter.

As with all studies that investigate aspects of high-grade glioma behavior, there are certain limitations to this study, which should be highlighted, such as the heterogeneous patient sample with respect to both the tumor site and the previous neurosurgical intervention, i.e., some patients had complete resections, some had partial resections, and some only stereotactic biopsies. However, this should not bias the results, since hemispherically averaged FA (with a threshold of 0.2) is not influenced by the extent of the resection and the remaining tumor mass. Another limitation is the number of patients with longitudinal DTI scans, which has to be seen in context that high-grade glioma exhibits aggressive courses with often short time periods of stable disease, and, thus, we included only longitudinal studies of patients without any evidence of tumor progression. Furthermore, the patient sample could have been also more homogeneous with respect to other cancer-specific therapy, such as chemotherapy or targeted-therapy, which was not taken into account at all. As no neuropsychological assessments in the three patients with longitudinal MRI scans were provided, a correlation between long-term radiation therapy-induced white matter injuries and decline of cognitive functions could not be assessed. While a subclinical tumor progression that is not detectable by standard neuroimaging tools could not be completely excluded, the clinical assessment had to rely on a formal classification as “stable disease,” which was the case in all patients as a mandatory criterion for study inclusion. Finally, this DTI-based study was performed with a clinical scanner at 1.5 T, and, therefore, the gradient strength and the field strength do not reach optimized DTI protocols that are available at 3 T or even 7 T (15). As a consequence, this study has to deal with a lower overall signal-to-noise-ratio compared to optimized high-field DTI studies. However, whether this should be seen as a limitation or as a strength of our study design, given that this study might be considered as a pilot for future clinical tumor studies that could be easily performed in a conventional clinical set-up, is open to debate.

An alternative to the fixed ROI calculation would be the segmentation of regions from T2 scans. However, due to the thresholding of FA values at 0.2, it was highly unlikely that any tumor tissue contributed to the interhemispheric FA differences, and hence the main read-out of this study, the global interhemispheric differences, was independent of the shape of the local interhemispheric differences, which could be performed by spherical ROI calculations or by ROI calculations that originate from exact segmentation masks from T2 scans.

To our knowledge, this is the first study comparing patients with high-grade glioma with and without previous radiation therapy in a combined cross-sectional and longitudinal study design. The cross-sectional part was designed to confirm that the observed differences are not due to tumor-associated microstructural injuries. The longitudinal approach analyzed chronically progressive alterations of white matter integrity due to radiation therapy in three patients without any clinical or diagnostic evidence of tumor progression. Taken together, this indicates that DTI changes might be used as an indicator for white matter microstructural alterations caused by brain radiation therapy and that those white matter alterations progress over long time periods (up to 5 years in our cohort), which is in general agreement with the clinical observations of cognitive decline in patients after brain radiation therapy (4, 5). To further investigate this issue, longitudinal studies with larger patient samples and neuropsychological assessments, divided into different populations with respect to tumor site and extent of tumor resection, might evaluate the vulnerability of distinct brain regions with respect to progressive damage of white matter integrity.

## Ethics Statement

The local ethics committee of the University of Ulm approved the study (project #41/17).

## Agreement

All authors are accountable for all aspects of the work in ensuring that questions related to the accuracy or integrity of any part of the work are appropriately investigated and resolved.

## Author Contributions

RK: conception and design of the study, interpretation of the data, recruitment of studied population, data acquisition, data analysis, preparation of the manuscript, final approval of the version to be published. MG: substantial contribution to the analysis of the data, critical revision of the manuscript, final approval of the version to be published. M-AW: substantial contribution to the analysis of the data (revised manuscript), critical revision and proofreading of the manuscript, final approval of the version to be published. AL: substantial contribution to the interpretation of the data, critical revision of the manuscript, final approval of the version to be published. JK: conception and design of the study, interpretation of the data, critical revision of the manuscript, final approval of the version to be published. H-PM: conception and design of the study, interpretation of the data, data analysis, critical revision of the manuscript, final approval of the version to be published.

## Conflict of Interest Statement

The authors declare that the research was conducted in the absence of any commercial or financial relationships that could be construed as a potential conflict of interest.

## Supplementary Material

Figure S1 Interhemispheric differences in axial, mean, and radial diffusivity (D) of 13 controls, 10 patients before or without radiation therapy, and 11 patients after radiation therapy.
