# Supplementary figures and images for: Cerebral Microstructural Alterations after Radiation Therapy in High-Grade Glioma: A Diffusion Tensor Imaging-Based Study
Source: Front Neurol. 2017 Jun 15;8:286. doi: 10.3389/fneur.2017.00286 (PMC5471301; doi:10.3389/fneur.2017.00286)

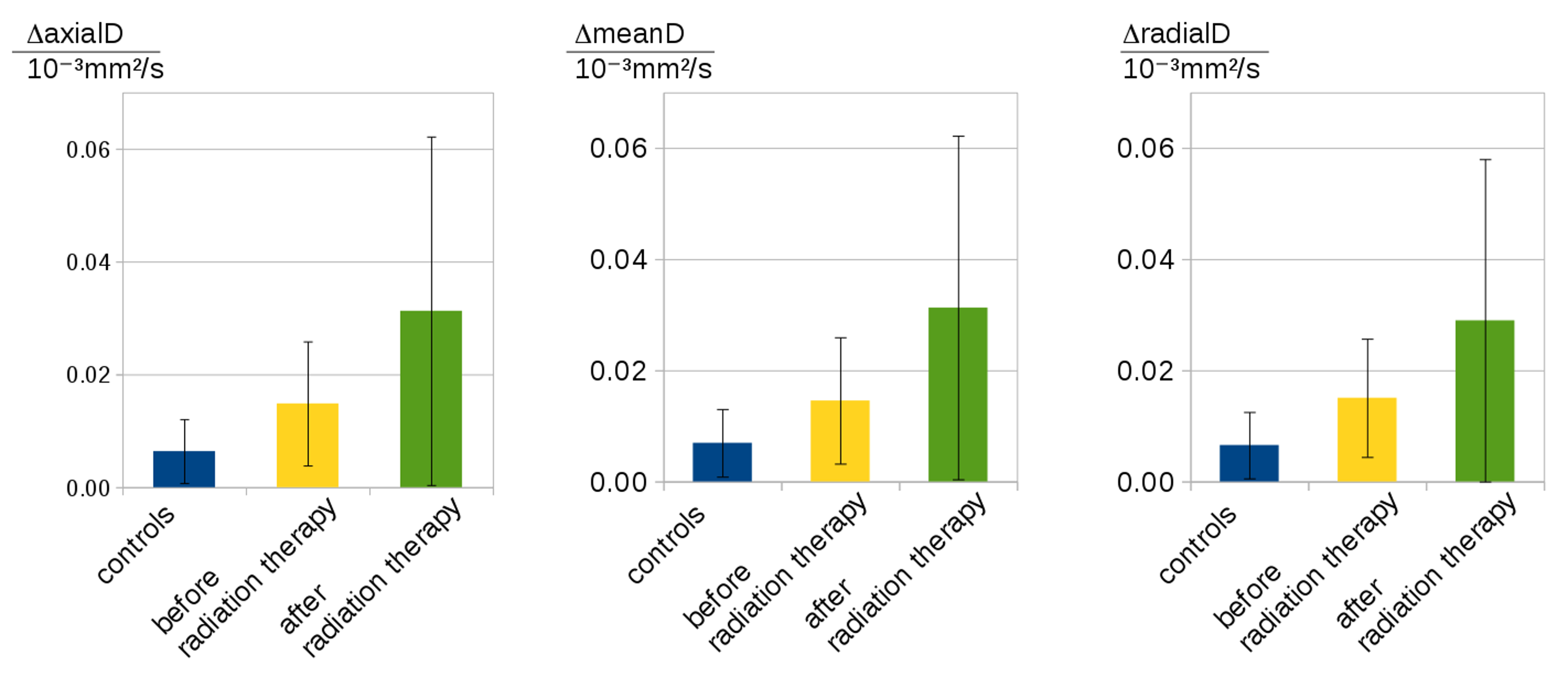

Supplement: Figure S1 — Interhemispheric differences in axial, mean, and radial diffusivity (D) of 13 controls, 10 patients before or without radiation therapy, and 11 patients after radiation therapy. [file image_1.tif]
